# The Pattern of Poisoning in Southwestern Region of Iran: Envenoming as the Major Cause

**Published:** 2012-08-25

**Authors:** Amir Jalali, Marzie Savari, Shaiesteh Dehdardargahi, Armita Azarpanah

**Affiliations:** 1 Department of Pharmacology and Toxicology, School of Pharmacy, Jundishapur University of Medical Sciences, Ahwaz, IR Iran; 2 Medical School, Persian Gulf International Division of Ahvaz Jundishapur University of Medical Sciences, Abadan, IR Iran; 3 Toxicology Research Center, Jundishapur University of Medical Sciences, Ahwaz, IR Iran

**Keywords:** Poisoning, Bites and Stings, Iran, Snake Bites

## Abstract

**Background:**

An analysis performed on the collected data from the Local Drug and Poison Information Centre (DPIC) of Jundishapur University revealed that stings are the main causes of poisonings with frequency of 56%, followed by drug poisoning in 31%, and chemical exposure poisoning in 5.5% in Khuzestan, the southwestern province of Iran.

**Objectives:**

The aim of the present study was to report the prevalence of poisoning in Khuzestan province referred to hospitals during the year 2007, on the basis of documents from the local Drug and Poison Information Centre (DPIC) and the main Khuzestan Hospitals Discharge Registry, to elucidate demographic trends of poisoning in this region.

**Materials and Methods:**

In the present study, 3258 cases of poisoning including 4.7% of all admissions to hospitals of Khuzestan during one year (2007) were investigated. Antidepressant drugs were the main-drug category inducing poisoning (24.37%). Others include sedative-hypnotics (19%), tricyclic antidepressants (TCA) (14.7%) and cardiovascular drugs (11.4%).

**Results:**

The research showed that most poisonings are occurred in autumn (29.6%) season. Besides the high poisoning rate of envenoming by animals in Khuzestan province, it seems that the pattern of poisoning is different with other Iran and worldwide regions.

**Conclusions:**

This may raise the attention of health service policy makers in Iran to establish a more effective diagnosis, management and implementing health policy services.

## 1. Background

Khuzestan province is located in the southwest of Iran with an approximate area of 64,746 sq.km. This province is the warmest region in Iran. According to the 2007 census, population of the province was estimated 4274979 comprising about 6.06% of the country population. Studies concerning genders proportion in Khuzestan province showed that male: female ratio was 51:49 during 1996-2007. Geographically, Khuzestan has surrounded by five other neighbor provinces named as Lorestan in the North, Ilam in the Northwest, Chahar Mahal va Bakhtiyari, and Kohkiloye va Boyerahmad in the East, and Bushehr in the Southeast. Based on statistical estimates, 35.5% of the population aged over 10, are involved in different economic activities and others including housewives, students, and people without a special job which organize the unemployed population of the province. Unemployment rate of Khuzestan province has been fluctuating between 16 and 17 percents recently.

The pattern of poisoning within a country depends on several factors such as the accessibility of various poisons, socio-economic status of the population, religious and cultural influences and drugs prescription manners (1-8) (Table 1). Different reports of poisoning in Iran indicate that the leading causes of poisoning or chemical injury in Iran are accidental cases followed by drugs poisoning and pesticides exposure, respectively (9-11).

**Table 1.** Characteristics of Available Literatures Including Poisoning Agents and Patients Groups

	No. of Cases	Country	Gender	Age Group	Duration, y	Leading Agent	Death rate, %
Al-Barrag and Farahat 2011 (1)	11964	Saudi Arabia	M/F	6-12	5	Drugs	
Jaiprakash *et al.* 2011 (2)	225	India	M	21-30	1	Organ phosphorous	12.8
Zhao *et al.* 2009 (3)	16506	China	F	20-29	10	Drugs	1.43
Thapa *et al.* 2008 (4)	148	Nepal	M	21-30	1	Organ phosphorous	
Malangu, 2008 (5)	276	Uganda	M	20-29	6	Organ phosphorous	1.4
Nurul Islam and Islam, 2003 (6)	2534	Bangladesh	M	13-24	10	Organ phosphorous	10.8
Laminpaa *et al.* 1993 (7)	114519	Finland	M	< 6 Year	2	Psychiatric drugs	0.7
Senanayake and Karalliedde, 1988 (8)	179	Srilanka	M	< 30 Year	3	Organ phosphorous	16

## 2. Objectives

The aim of the present study was to report the prevalence of poisoning in Khuzestan province referred to hospitals during the year 2007, on the basis of documents from the local Drug and Poison Information Centre (DPIC) and the main Khuzestan Hospitals Discharge Registry, to elucidate demographic trends of poisoning in this region. This study will certainly compare the probability of poisoning occurrence in Khuzestan province with other parts of Iran and the worldwide prevalence in order to provide some preventative solutions concerning with poisoning burden.

## 3. Patients and Methods

This analytical retrospective cross-sectional study was carried out on the basis of data collected from the local DPIC, Jundishapur University of Medical Sciences and the main Khuzestan Hospitals Discharge Registering during the year 2007. This project was authorized by the national DPIC of health ministry. A questionnaire about poisoning information was prepared and distributed among physicians in provincial hospitals to collect poisoning patients data. A total of 3258 poisoning cases were overviewed in the present study. In addition, all cases admitted to emergency departments with poisoning diagnosis were investigated, even individuals with no obvious symptoms and directly discharged were studied. It should be mentioned that all the diagnoses were based on patients medical history, physical examinations, and toxicology laboratory evaluations. It should be noted that poison, by definition, is a substance be able to cause damages or result in dysfunctions in the body organs and poisoning is identified as an inadvertent or excessive intake of a medicine, a non-drug accident, a drug or a non-drug for suicidal purposes, or a non-drug poisoning with unknown purpose (12). The variables in the present study were causes of poisoning, age, sex, name of poisonous substances; severity and outcome. This research was approved by the Jundishapur University of medical sciences Ethical Committee, Ahvaz, Iran.

### 3.1. Statistical Analysis

The data were analyzed using SPSS 16.0. All results were presented as numbers and percentages.

## 4. Results

### 4.1. Demographic Properties of the Poisoned Patients

Analysis of questionnaire data on 3258 poisoned patients who had attended Emergency Departments of the main hospitals of Khuzestan province during one year study period (2007) showed that there were 1650 (51%) males and 1608 (49%) females. The majority of poisoning were remarkably found in the age group of 18-30 years (34%) followed by 12-18 with 19% and 13.77% of the patients were below 12 years of age (Table 2).

**Table 2.** Distribution of Poisoning by the Age Groups

Age Group	Number of Cases	Percent, %
0-2	123	3.77
2-12	334	10
12-18	617	19
18-30	1114	34
30-40	502	15
40-60	397	12
>60	171	5
Total	3258	100

### 4.2. Poisoning Agents

**Figure 1.**
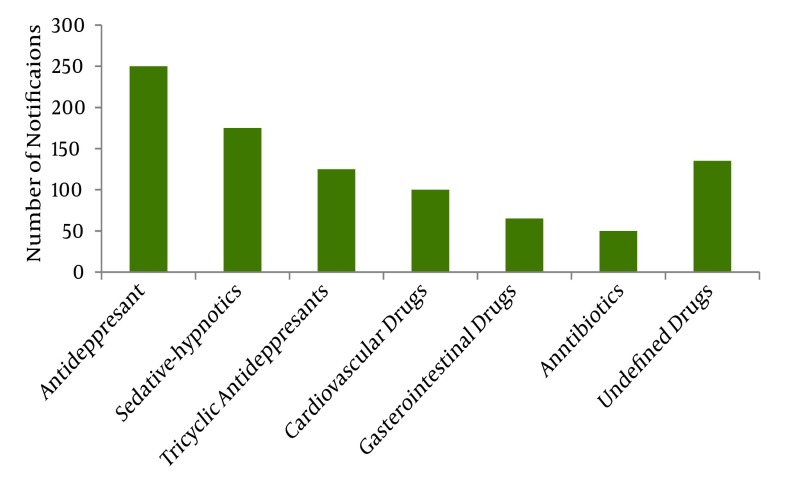
Number of reported Poisoning Cases in Khuzestan According to the Drug Groups throughout the Year 2007.

In the present study, the most frequent causes of intoxication were envenoming by a venomous animal (56%) and drug poisoning (31%). Envenoming was majorly caused by venomous animals such as scorpions, snakes and spiders. The study indicates that 1791 cases of poisoning were due to animal bites and stings, including 312 (17%) poisoning belonging to snake bites, 18 cases (0.01%) belong to spiders and other unrecognized venomous animals and the 1469 remaining cases (82%) were due to scorpions envenoming. Chemical poisoning comprised 5.5% of recorded cases while organophosphorous poisoning accounted for 59(1.8%) of cases (Table 3). The drug poisoning was remarkably due to antidepressants with 247 cases (24.3%), followed by sedative-hypnotics in 193 cases (19%), tricyclic antidepressants (TCA) in 149 cases (14.7%), cardiovascular drugs in 116 cases (11.4%), gastrointestinal agents in 74 cases (7%), antibiotics in 66 cases (6%) and undefined drugs in 169 cases (17%) (Figure 1).

**Table 3.** Causes of Hospitalization due to Poisoning (n = 3258)

Cause of Poisoning	Number of Cases	Percent, %
Food poisoning	120	3.7
Plant poisoning	36	1.1
Drug poisoning	1014	31.6
Chemical poisoning	179	5.5
Envenomation by venomous animals	1799	56.2
Organophosphor &insecticides	59	1.8
Opiate	51	1.6
Total number	3258	100

### 4.3. Admission Status

Full recovery was observed in 82.8% of cases while partial remedy was occurred in 13.1% of the cases. Poisoning-induced damages happened in 2.7% of the cases. Among all admitted patients, the mortality rate was 0.3% (10 cases) (Table 4).

**Table 4.** Different Consequences of Poisonings (n = 3258)

Condition	Number of Cases	Percent,%
Full remedy	2684	82.38
Partial remedy	427	13.10
Poisoning caused injuries	49	1.50
Referred to other centers	88	2.70
Death	10	0.3

### 4.4. Routes of Poisoning

Ingestion with suicidal intentions was a common pattern of poisoning. In the present investigation, 3.7% of poisoning cases occurred through oral ingestion.

### 4.5. Time of Referral

**Figure 2.**
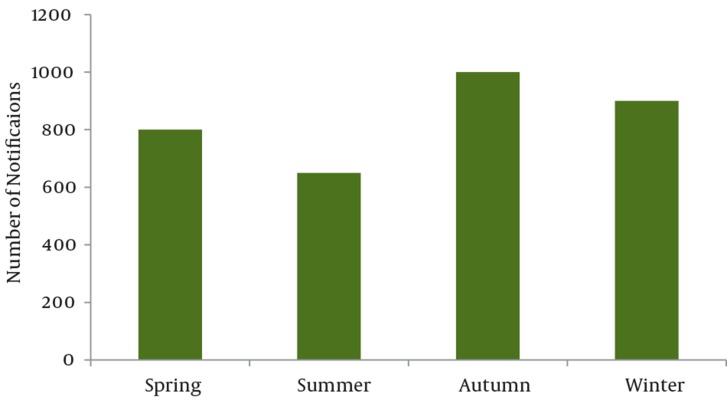
Number of reported poisoning cases according to seasonality throughout the year 2007.

Data concerning with the time interval between poisoning and referral to emergency departments of hospitals were only reported by 1890 cases. 65.4% of the cases were referred to the hospital in less than three hours after the poisoning, 24.8% three to four hours, and 9.8% more than four hours. The majority of poisoning occurred during autumn (29.6% of cases) (Figure 2).

## 5. Discussion

The main cause of poisoning in southwest of Iran, Khuzestan belongs to venomous animals sting. It was observed in this study for the first time that the leading causes of poisoning are stings. So far this finding is different from other existing reports from other parts of Iran and the world (Table 1). The results highlight the importance of venomous animal stings as a potential poisoning agent especially in the 18-30 age groups. With respect to age groups, it is shown that the age group below 30 years old has faced 53% of all poisonings. It is also seen that the 18-30 age group is more liable to poisoning. This is similar to other studies related to poisoning in Tehran and northern parts of Iran (13, 14). A possible explanation could be that younger individuals have higher levels of activities and consequently more exposure rates.

Previous retrospective studies and available data showed that scorpion’s sting is one of the most common causes of hospital referrals in hot seasons. Among different types of scorpions, H. lepturus envenoming which is highly lethal is very common (15-17). So far 24 species of scorpions have been identified in Iran, which from those 12 species were found in Khuzestan (18). The medically important species found in this region are Androctonus crassicauda, Mesobuthus eupeus and Hemiscorpius lepturus. While the first two belong to the Buthidae family, the latter is a member of Hemiscorpiidae, which is the most venomous of all types of scorpions in Iran (19, 20). The most cases of stung patients have been reported from Mexico worldwide (21). The prevalence rate of scorpion stings was previously reported 3.1/1000 in habitants of Khuzestan. In Shahabzadeh study a number of 12150 scorpion stung patients in Khuzestan were reported following a stratified cluster random sampling method in 2003 (22). Generally About 60% of all stings in Iran occur in this province. This high rate is followed by the neighborhood provinces, Kohkiloye va Boyerahmad and Ilam (23). dry and mid-dry climate of Khuzestan presents an appropriate environment for the Arthropods including venomous animals mainly scorpions. Venomous snake bites are common and also deadly emergency during the warm months. Venomous snakes in this area mainly have hemotoxic and/or neurotoxic venoms. changes occurred during the 8 years of war with Iraq resulted in an increase of population, immigration, construction works in rural regions and cities in neighborhood using natural materials such as stone. These high risk conditions and environmental changes have enhanced sting rates especially by scorpions (24). It is worth noting that stings were occurred in all year round. However, the majority of the venomous animals envenoming took place in the warmest months of the year.

The majority of poisoning cases were happened during the 3rd quarter of the year. The majority of referrals to clinical centers due to drug poisoning were in autumn. Seasonality effects on poisoning distribution throughout the year have been reported previously. It was described that poisoning in northern of Iran was mainly due to organphosphorous during the spring and summer because of abundant use of these compounds (25). The higher rate of poisoning which occurs in autumn may be attributed to more drugs consumption following weather changes. It was noticed that the average number of drug items per prescription in Tehran, the capital city of Iran was 3.6 (26). This average was 3.80 and higher in Khuzestan (according to available data at local DPIC). Despite the fact this average was 1 in developed countries and 1.2 to 1.4 in developing countries including Iran (27). So, available data show that prescriptions and drugs usage in these parts of the world does not obey the academic and standard principals. One of the main causes of poisoning among drug groups was antibiotics. This result was expectable because more than a half of prescriptions (50-60%) in the past few years in Khuzestan have contained at least an antibiotic drug.

The opiate poisoning allocated only 1.6% of the cases. In Tehran, opium was reported as the main agents responsible for intoxication especially in fatal cases (10). The results of the present study show that opiates haven’t been the main cause of poisoning. However it is possible that the majority of Opium-addicted patients might not refer to the hospitals after poisoning. Moreover it is also obvious that Khuzestan is not located within the transferring pathway from producing countries like Afghanistan and Central Asia to the Europe. Full remedy of 82% of cases in Khuzestan hospitals is one of the merits of managing intoxicated patients vs. other investigations such as Karbakhsh and Salehian-Zandi, 2007 (10).

According to our study, males and females were both affected similarly. This result was not significantly different in compared with male to female ratio of Khuzestan population reported by Iranian Statistical Center in 2007. This finding is not well-matched to other studies (Table 1). It is well known that the comparison of different studies in poisoning cases is difficult because types of poisoning vary according to different regions and causes. But it is a common belief that females are most vulnerable to commit suicide by taking different poisons mainly drugs. As showed, attempted suicide was not very common and only compromising 4.3% of all cases (141 poisoning cases). The major cause of poisoning was sting and it affected males more in contrast to females (more than 60% of stings were in males), so this result may be anticipated. The poisoning via ingestion was not frequent. This data was against reported studies in different regions such as Asia-Pacific (28), Tehran (29) and New Zealand (30).

Khuzestan is an agricultural area. Hence, the pesticides are used and accessible widely. Therefore it seems easy to conclude that poisoning with these compounds is likely to be one of the main causes of poisoning. However, the pesticide poisoning from occupational, accidental and intentional exposures is anticipated (31). The data showed that only 1.8% of the cases were pesticide poisoning. This pattern is not in concordance with previous studies in Iran such as Moghadamnia and Abdollahi, 1998 (25), but similar to developing countries (32). Moghadamnia and Abdollahi reported that the majority of poisoning cases in northern Iran was due to fertilizers and pesticides (25).

Drugs poisonings and H.lepturus sting from non-drug reasons were more prevalent in cases which required longer stay at Khuzestan hospitals. . Unluckily there is not an available record for drug poisoning reasons. However this is understandable the majority of drug poisonings may be suicidal intentions, whereas non-drug poisonings occur more commonly in small children. The overall mortality in this study was 0.3% (10 cases) out of which 5, 3 and 2 cases were seen in patients with organphosphorous, drugs and stings poisoning, respectively. So it is reasonable to elucidate that the organphosphorous, drugs and stings by far are the most common causes of hospitalized poisoning death. The typical causes of fatal poisoning vary greatly from one part to another in a country and even from a country to another. A performed study in the Loghman Hospital (Tehran) in 1996 showed that, out of 16.531 cases, the overall mortality rate was 1.08% but major causes were different from the present study (33). The mortality rate in this study was less than other reports in Iran (13, 14), Turkey (0.7%) and India (33-35). Poisoning does not account for an important category of death causes in Khuzestan. When interpreting the low mortality rate of poisoning, it is important to consider that many cases especially stings with mild or moderate poisoning presentations are not referred to medical centers, but are managed at home; conversely, some death cases due to intoxication may be directly referred to medicolegal departments and so are not included in this study. Furthermore, the presented data (Table 1) indicates that organphosphorous pesticides are the predominant cause of death. This poisoning agent is responsible for the majority of deaths (28). So the mortality rate in Khuzestan, where organphosphorous poisoning is relatively uncommon (Table 3), doesn’t look like to have a significant difference with the presented value.

Evaluation of our poisoned patients during a year showed that pharmaceutical agents account for a considerable part of substances causing poisoning (31.6% of all cases). The main drug-groups causing poisoning were CNS-acting drugs. Regardless of the type of poisoning (intentional or accidental), drug agents were responsible for the second cause of substances involved in poisoning. This finding is consistent with previous studies in different age groups in Iran (13, 14, 24), and also in other parts of the world (1-8). The prevalence of drug poisoning in below 12 years of age group was greatly different from Jaraczewska et al. 1997 report (36). Fortunately, one of the drug groups was antibiotics. This group of drugs is less toxic than antidepressant drugs mainly TCAs and sedative-hypnotics. The most common route of entry was oral and is in accordance with available reports (13, 37). The low rate of fatal cases in this study may be attributed to the low opiate rate of intoxication. The main agents responsible for intoxication in fatal cases were opioids and opiate products in other studies(10). The low rate of mortality in this study highlights the importance of opium as a potentially life threatening agent.

### 5.1. Limitations of the Study

In the southwestern part of Iran, Khuzestan, envenoming by venomous animals is the most common cause of poisoning and hospital referrals. The main limitation is that the numbers do not cover those with serious poisoning requiring referral and hospitalization, so, these results don’t reflect the whole poisoning events throughout the year 2007. In addition, the rate of hospitalization was reported at the time of discharge, but not in remote time so; these results may trigger some inaccuracies.

Besides the high poisoning rate of envenoming by animals in Khuzestan province, it seems that the pattern of poisoning is different with other Iran and worldwide regions. This may raise the attention of health service policy makers in Iran to establish a more effective diagnosis, management and implementing health policy services. In conclusion, there is an urgent condition to receive high numbers of antivenom agents in regions like Khuzestan where Viperidae envenoming mainly Echis carinatus and scorpion stings mainly H. lepturus are a major public health burden.
